# Enhanced Thermoelectric Properties of Misfit Bi_2_Sr_2-x_Ca_x_Co_2_O_y_: Isovalent Substitutions and Selective Phonon Scattering

**DOI:** 10.3390/ma16041413

**Published:** 2023-02-08

**Authors:** Arindom Chatterjee, Ananya Banik, Alexandros El Sachat, José Manuel Caicedo Roque, Jessica Padilla-Pantoja, Clivia M. Sotomayor Torres, Kanishka Biswas, José Santiso, Emigdio Chavez-Angel

**Affiliations:** 1 Catalan Institute of Nanoscience and Nanotechnology (ICN2), CSIC and BIST, Campus Universitat Autonoma de Barcelona (UAB), Bellaterra, 08193 Barcelona, Spain; 2 New Chemistry Unit, School of Advanced Materials, Jawaharlal Nehru Centre for Advanced Scientific Research (JNCASR), Jakkur, Bangalore 06484, India; 3 ICREA—Catalan Institute for Research and Advanced Studies, 08010 Barcelona, Spain

**Keywords:** misfit cobaltates, thermoelectric properties, misfit-layer, isovalent substitutions

## Abstract

Layered Bi-misfit cobaltates, such as Bi_2_Sr_2_Co_2_O_y_, are the natural superlattice of an electrically insulating rocksalt (RS) type Bi_2_Sr_2_O_4_ layer and electrically conducting CoO_2_ layer, stacked along the crystallographic c-axis. RS and CoO_2_ layers are related through charge compensation reactions (or charge transfer). Therefore, thermoelectric transport properties are affected when doping or substitution is carried out in the RS layer. In this work, we have shown improved thermoelectric properties of spark plasma sintered Bi_2_Sr_2-x_Ca_x_Co_2_O_y_ alloys (x = 0, 0.3 and 0.5). The substitution of Ca atoms affects the thermal properties by introducing point-defect phonon scattering, while the electronic conductivity and thermopower remain unaltered.

## 1. Introduction

The search for renewable energy sources and energy recovery methods is an active area of research. Direct conversion of waste heat into electricity using the thermoelectric Seebeck effect has received attention in recent years [1]. High efficiency in a thermoelectric module requires a high “figure of merit” (zT), which is a balance between power factor and thermal conductivity (zT = (σ*S*^2^)T/k, where σ, S, k, and T are the electronic conductivity, Seebeck coefficient, thermal conductivity, and mean operational temperature, respectively). For high TE efficiency, a high zT is mandatory, i.e., a large power factor (PF = σS^2^) and low k are essential elements [2]. Recent studies in layered materials based on bismuth telluride, silicene films, tin selenide, and multilayer structures of dissimilar 2D materials have demonstrated zT values above 2 at room temperature [3,4,5,6]. However, oxide materials will be an alternative solution for high-temperature thermoelectric application because of their chemical stability at high temperatures, non-toxicity, and less expensive constituent elements [7,8,9,10,11].

Soon after the discovery of a large Seebeck coefficient (~100 μV/K) and relatively high electrical conductivity (~5000 S/cm) in Na_0.5_CoO_2_ (NCO) at room temperature, the layered cobaltates are considered as a promising class of compounds for thermoelectric energy conversion [12]. Aside from cobaltates, BiCuSeO oxyselenides have also been identified as potential thermoelectric materials, showing a zT close to 1 [13] and Seebeck coefficient around 500 μV/K [14]. The misfit cobaltate [15] Bi_2_Sr_2_Co_2_O_y_ (BSCO) is a *p*-type thermoelectric material and has shown a figure of merit (zT) > 1 at 1000 K [16].

NCO and BSCO share a common CdI_2_-type CoO_2_ layer in their crystal structures [17]. The CoO_2_ layers in BSCO are separated by the rocksalt (RS)-type Bi_2_Sr_2_O_4-δ_ layers. RS and CoO2 layers do not fit along the crystallographic b-axis (because the b parameter of RS > b-parameter of CoO2), hence the name misfit [18]. The incommensurate nature of the crystal structure results in the ultralow cross plane [19,20] and low in-plane [21] thermal conductivity, which is one of the key factors responsible for the high zT.

It is well known that the CoO_2_ layer governs the electronic transport properties of misfit cobaltates because the RS layer is electrically insulating in nature [17]. Nevertheless, these two layers are intimately related to each other through charge transfer [22]. Given that NCO and BSCO share common triangular CoO_2_ layers, their electronic structure near Fermi energy (E_F_) are quite similar [23]. At room temperature, σ of BSCO is lower (~330 S/cm) than in NCO, but S remains high for both compounds (100–120 μV/K) [24]. At high Na doping in NCO, σ and S remained very high [25], whereas Pb doping in BSCO also showed improved σ, and hence improved PF [26,27]. However, σ decreased drastically in Bi_2_M_2_Co_2_O_y_ (M = Ba, Sr and Ca) when Ba was substituted by Sr or Ca [28]. Note that even though the RS layer is electrically insulating, doping or substitution in the RS layer produced significant change in the transport properties. In fact, the electronic band structure near the Fermi energy (E_F_) was significantly affected (transfer of spectral weight from the broad itinerant band to an incoherent regime was observed) in Bi_2_M_2_Co_2_O_y_ (M = Ba, Sr, and Ca) when Ba was replaced by Sr or Ca [23]. Therefore, RS layers provide tunable possibilities to understand the charge transfer mechanism and optimize conditions to achieve the best thermoelectric performance.

To this aim, several strategies were employed in the single crystal and polycrystals of BSCO. For example, an improved zT (0.023 at 300 K) was reported in Pb-substituted BSCO single crystals [26]. A different set of single crystals of composition Bi_2.3-x_Pb_x_Sr_2.6_Co_2_O_y_ (0 ≤ x ≤ 0.44) showed enhanced *S* and σ upon Pb substitution in the RS layer (PF ~ 9 μW/cm.K^2^ at 100 K) [27]. Polycrystalline Bi_2_Sr_2_Co_2_O_y_ showed high-temperature stability and zT ~ 0.2 at 1000 K, however this was very much dependent on the composition [29]. Ca-substituted polycrystalline sintered BSCO prepared by the sol-gel method showed lower zT ~ 0.007 at 300 K [30,31]. Modifying the sintering conditions, zT = 0.05 in BSCO was achieved at 150 K [32]. To the best of our knowledge, high-temperature thermoelectric properties of Bi_2_Sr_2-x_Ca_x_Co_2_O_y_ (x = 0, 0.3 and 0.5) samples have not been reported yet.

In this work, we investigate the temperature dependence (340 < T < 750 K) of the thermoelectric properties of spark plasma sintered (SPS) parent Bi_2_Sr_2_Co_2_O_y_ and their Bi_2_Sr_2-x_Ca_x_Co_2_O_y_ alloys (x = 0.3 and 0.5). Although isovalent ion substitutions (Sr^+2^ by Ca^+2^) did not significantly impact the electronic properties, a strong decrease in κ was found. This result reflects the selective phonon scattering that occurred by point defects in the misfit cobaltates, which caused an improved zT ~ 0.02 at 300 K and 0.09 at 740 K in Bi_2_Sr_2-x_Ca_x_Co_2_O_y_ alloys (within x = 0.3 and 0.5).

## 2. Experimental Methods

Synthesis: Bi_2_Sr_2-x_Ca_x_Co_2_O_y_ (x = 0, 0.3 and 0.5) polycrystalline pellets were synthesized by conventional high temperature solid-state reactions [29]. A stoichiometric mixture of Co_3_O_4_, Bi_2_O_3_, SrCO_3_, and CaCO_3_ were mixed in an agate mortar to obtain a homogeneous powder. The powder was pressed using a stainless-steel die into a pellet under uniaxial pressure. The pellet was slowly heated up to 1070 K and calcined for 40 h at the same temperature in air and then slowly cooled down to room temperature. The calcined pellet was ground into a fine powder and then pressed again into a pellet in uniaxial pressure for 30 min to achieve dense pellets. Then, the pellets were placed into a tubular furnace and sintered at 1160 K for 20 h at 100 sccm pure oxygen flow. Samples were prepared by the SPS method at 920 K in a uniaxial pressure of 3.1 kN (~40 MPa) to achieve high density (~94.3%) for thermoelectric transport measurement.

Characterization: Polycrystalline sample quality and θ-2θ diffraction patterns were checked by X-ray diffraction technique at room temperature using a Malvern-Panalytical X’pert Pro MRD (multipurpose X-ray diffractometer, Malvern, UK) tuned at Cu K-alpha radiation of wavelength 1.54598 Å. Out-of-plane lattice parameters were calculated from (005) reflections. X-ray photoelectron spectroscopy (XPS) measurements were carried out at room temperature in order to identify the elements and their oxidation states.

The Raman spectra were recorded by a T64000 Raman spectrometer manufactured by HORIBA Jobin Yvon (Chilly-Mazarin, France) in single grating mode with 2400 lines and a spectral resolution better than 0.4 cm^−1^. The measurements were performed by focusing a diode laser (532 nm) onto the sample with a 100× microscope objective. The power of the laser was kept as low as possible (~0.5 mW) to avoid any possible damage from self-heating of the samples.

Transport properties: The Seebeck effect and electronic conductivity measurements were carried out simultaneously from 350 to 750 K in a LINSEIS instrument (in a 1 atm helium pressure). Thermal diffusivity measurements were carried out in a NETZSCH LFA instrument (with continuous N_2_ gas flow). Pellets were cut into 2 × 2.5 × 8 mm^3^ and 6 × 6 × 2 mm^3^ dimensions for the Seebeck effect and thermal diffusivity measurements, respectively. Thermal conductivity was calculated from the relation, $\kappa_{total}=DC_{p}d$, where D, C_p_, and d are thermal diffusivities, specific heat capacity, and density of the pellet, respectively. It is important to note that due to the polycrystalline nature of the samples, there are no preferred orientations in the crystal structure. Thus, we did not find anisotropies in the thermal and electronic properties of synthesized samples. The measurement of thermal conductivity is subject to an uncertainty of approximately 5%, which is dependent on the chosen characterization method and the underlying assumptions made in the modeling of thermal properties. The three-omega technique is widely regarded as a highly effective method for determining thermal conductivity, and it has been shown to achieve uncertainties below 1% when specific experimental conditions are met [33]. In comparison, the flash method is associated with a relatively low uncertainty of around 3 to 5% for the estimation of thermal diffusivity [34].

## 3. Results and Discussion

### 3.1. Structural and Elementary Characterization

**Figure 1:**
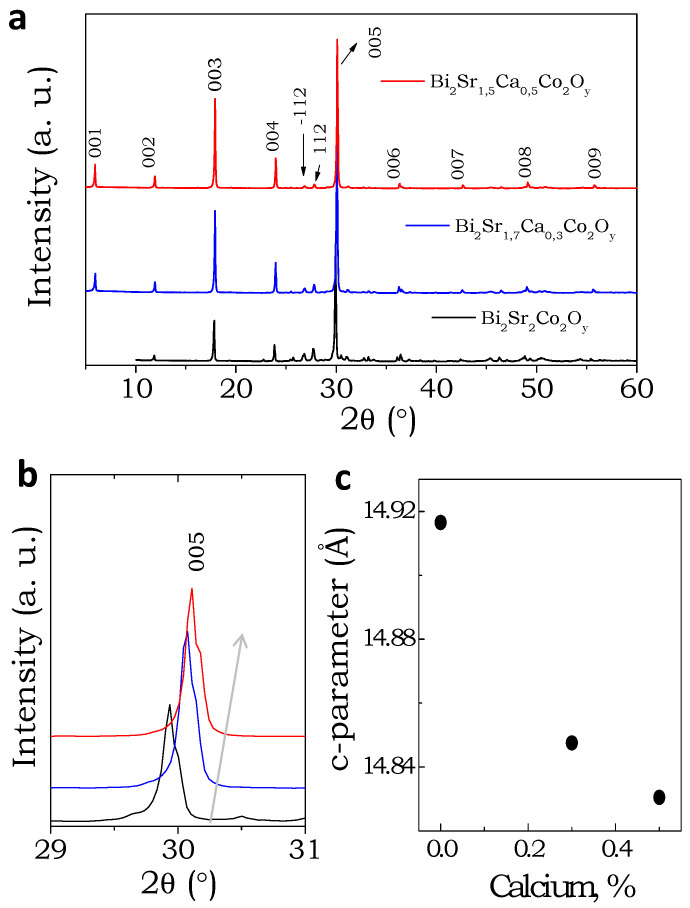
X-ray diffraction (XRD) patterns. (**a**) Powder X-ray diffraction patterns of Bi_2_Sr_2-x_Ca_x_Co_2_Oy (x = 0, 0.3 and 0.5) samples, (**b**) shift of the 005 reflections in 2θ upon substitution, and (**c**) change of the c-parameter with increasing calcium content.

Figure 1 depicts the powder X-ray diffraction (XRD) patterns of the Bi_2_Sr_2-x_Ca_x_Co_2_O_y_ (x = 0, 0.3 and 0.5) samples. The power XRD patterns are similar to the polycrystalline samples synthesized by others [26]. No impurity phases were detected within the detection limit of the diffractometer. A systematic shift of (005) reflection in 2θ position can be observed with increasing Ca content, which indicates that the out-of-plane cell parameters decreases with increasing Ca content. As the ionic radii of Ca^+2^ ion is smaller than Sr^+2^ ions, the unit cell undergoes a systematic contraction [35]. The change in the c-parameter upon substitution confirms the incorporation of Ca atom in the BSCO unit cell.

**Figure 2:**
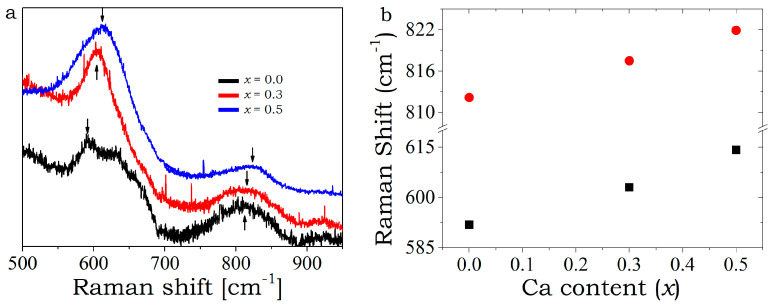
(**a**) Raman spectra of Bi_2_Sr_2-x_Ca_x_Co_2_Oy (x = 0, 0.3 and 0.5) samples at 300 K cm^−1^. (**b**) Peak positions of Raman band marked with arrows.

To prove the incorporation of calcium in the BSCO lattice, Raman spectra measurements were also carried out at room temperature. Figure 2 represents the recorded Raman spectra of Bi_2_Sr_2-x_Ca_x_Co_2_O_y_ (x = 0, 0.3 and 0.5) samples from 500 to 950 cm^−1^ at 300 K. Two prominent phonon peaks can be observed for all samples at ~618 cm^−1^ and ~815 cm^−1^. The peaks at 618 cm^−1^ can be assigned to A_1g_ modes, comparing with the Raman spectra of single crystals of similar composition [19]. The A_1g_ modes represent the out-of-plane vibrational mode of oxygen atom. A slight right shift in the position of Raman peak in Figure 2b can be observed with increasing Ca content. This can be explained by the increase in bond strength of the Ca-O bond, with respect to the Sr-O bond.

**Figure 3:**
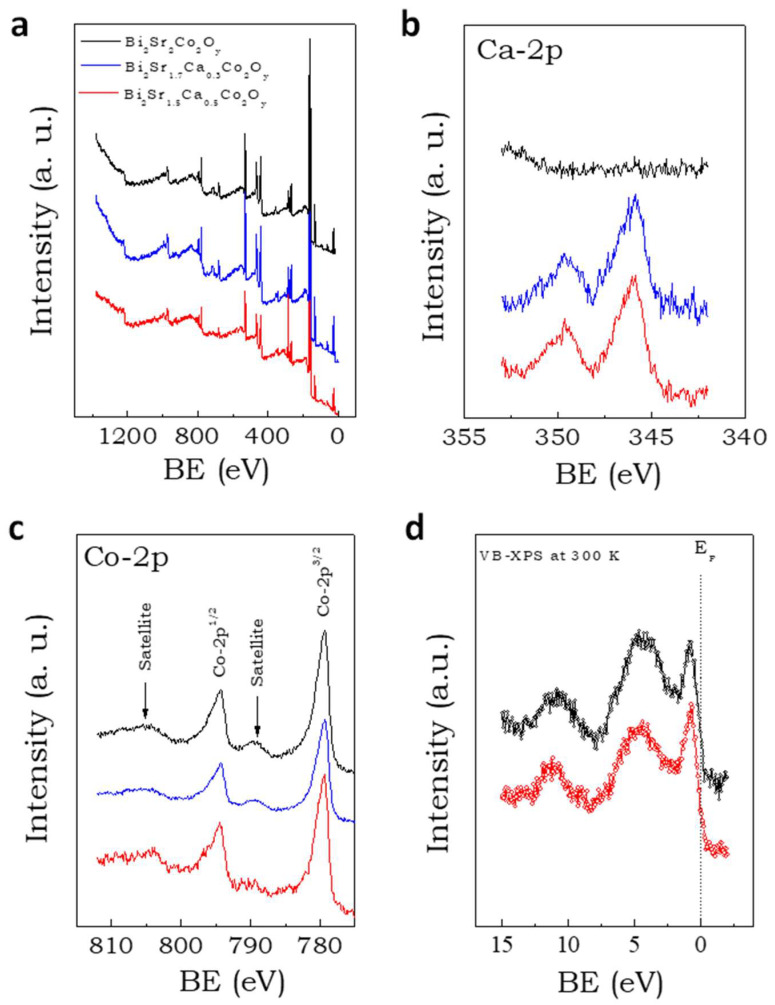
X-ray photoelectron spectroscopy. (**a**) Overview of the XPS spectra of Bi_2_Sr_2-x_Ca_x_Co_2_Oy for x = 0 (black line), 0.3 (blue) and 0.5 (red). (**b**) High-resolution core level XPS spectra of Ca-2p for x = 0 (black line), 0.3 (blue) and 0.5 (red), (**c**) high-resolution core level XPS of Co-2p for x = 0 (black line), 0.3 (blue) and 0.5 (red). (**d**) valence band-XPS spectra of Bi_2_Sr_2-x_Ca_x_Co_2_Oy for x = 0.3 (black) and 0.5 (red) samples.

Element detection and oxidation states of cobalt ions were obtained from XPS, as shown in Figure 3. The overall spectrum contains prominent peaks of Bi, Sr, Ca, Co, and O (see Figure 3a). The high-resolution core level XPS spectrum of Ca-2p is depicted in Figure 3b. As can be seen in Figure 3b, the parent composition did not contain Ca, while the alloys showed prominent peaks of Ca-2p. The high-resolution core level spectrum of Co-2p of Bi_2_Sr_2-x_Ca_x_Co_2_O_y_ (x = 0, 0.3 and 0.5) compositions is depicted in Figure 3c. The Co-2p spectrum was split into two components—Co 2p^3/2^ and Co 2p^1/2^—due to strong spin-orbit coupling at an intensity ratio of ~2:1. The appearance of the satellite peaks from both parts of the 2p components at higher binding energies to the main peaks indicates the presence of mixed valence cobalt ions. Misfit cobaltates showed satellite peaks due to the presence of Co^+3^/Co^+4^ pair. The line shape of the Co-2p core-level spectrum indicates the presence of low-spin (LS) Co^+3^ ions. Valence band spectrums of the parent and alloys are depicted in Figure 3d. They are very similar to those obtained in single crystals [36]. The density of states (DOS) near the Fermi energy (E_F_) is dominated by the hybrid molecular orbitals of Co-3d and O-2p. The sharp peak around 1.5 eV is due to the presence of LS Co^+3^ ions (t_2g_^6^e_g_^0^). A small amount of DOS is embedded in the E_F_; therefore, the compounds were expected to show metallic behavior in their transport properties.

### 3.2. Thermoelectric Transport Properties

**Figure 4:**
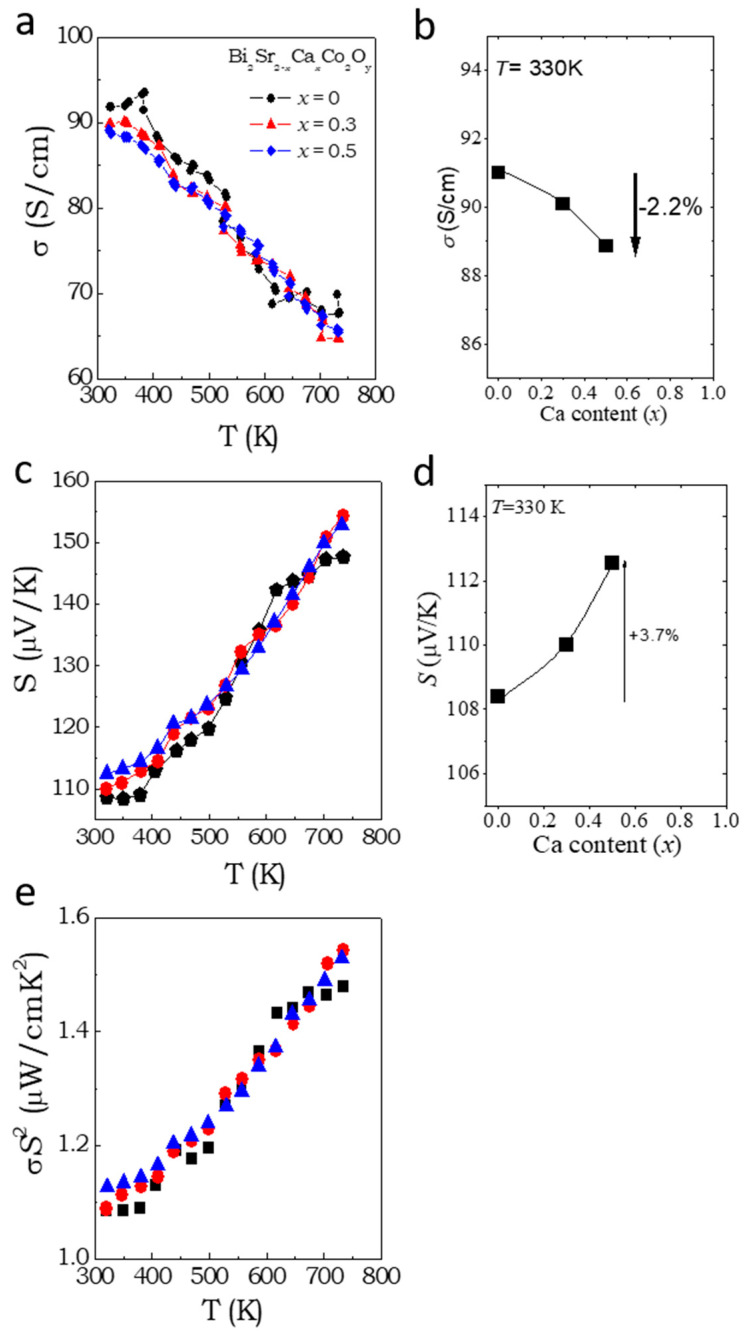
Electronic conductivity and thermoelectric Seebeck effect. Temperature dependent (**a**) electronic conductivity, (**c**) thermoelectric power, and (**e**) power factor of Bi_2_Sr_2-x_Ca_x_Co_2_O_y_ (x = 0, 0.3 and 0.5) samples. Effect of calcium substitution on (**b**) electronic conductivity and (**d**) Seebeck coefficient at 330 K.

Temperature dependence of S and σ of Bi_2_Sr_2-x_Ca_x_Co_2_O_y_ (x = 0, 0.3 and 0.5) samples are depicted in Figure 4. σ is ~90 S/cm at 340 K for the parent composition (see in Figure 4a). We found that σ decreases linearly with increasing temperature from 340–750 K, which is typically observed in metallic conductors. This result is consistent with the previous valence band XPS measurements, where a small amount of DOS was found near E_F_ (see Figure 3d). The positive value of S for the parent composition and their alloys indicates the *p*-type nature of the compounds (Figure 4b). This also points out the presence of Co^+3^/Co^+4^ mixed valence states in the CoO_2_ layer. The S reached a value of +108 μV/K for the parent composition and increased linearly with increasing temperature up to 145 μV/K at 750 K. This is a typical metallic-like behavior.

The influence of calcium substitution on the electronic conductivity and Seebeck coefficient of the BSCO lattice is displayed in Figure 4b,d. The conductivity decreases by a small margin (2.2% drop within the range of x = 0–0.5 of Ca substitution) while the Seebeck coefficient increases slightly (3.7% increase within x = 0–0.5). These changes are driven by variations in the concentration of *p*-type charge carriers caused by Ca^2+^ substitution for Sr^2+^ ions, which is isovalent and should not affect carrier concentration. However, the valence of cobalt ions in the CoO_2_ layer, which controls the carrier concentration, is linked to the misfit ratio in BSCO single crystals, as revealed in the literature [37]. Typically, in standard semiconductors or semimetals, the electronic conductivity and Seebeck coefficient exhibit opposing behavior with doping or substitution, as observed in our samples. However, in the literature [37], it was shown that the valence of cobalt ions in the CoO_2_ layer is related to the misfit ratio in the single crystals of BSCO, as follows;

(1) $$v_{\mathrm{Co}}=4-\frac{\alpha }{q\;}\;;\;q=\frac{b_{\mathrm{RS}}}{b_{{\mathrm{CoO}}_{2}}}$$

where, $v_{\mathrm{Co}},\;\alpha \;,\;\mathrm{and}\;q$ are the valence of cobalt ion, charge of electrically insulating RS layer, and the misfit ratio of $b_{\mathrm{RS}}$ to $b_{{\mathrm{CoO}}_{2}}$ ($b_{\mathrm{RS}}$ and $b_{{\mathrm{CoO}}_{2}}$ are the b-lattice parameters of the rock salt type and CoO_2_ layer), respectively. We found that the value of “α” remained constant despite isovalent substitutions. Hence, the alteration in “v” “Co” is expected to be minimal due to the substitution of Ca for Ca^+2^ in Bi_2_Sr_2-x_Ca_x_Co_2_O_y_, within the range of x = 0.3 to 0.5. This may result from either decreased *p*-type carrier concentration in the CoO_2_ layer due to oxygen vacancy formation or minimal change in the misfit ratio. To uncover the dominant factor affecting the lattice parameters, we performed a calculation of the average in-plane lattice parameters of Bi2Sr_2-x_Ca_x_Co_2_O_y_ (x = 0, 0.3, and 0.5) using X-ray diffraction (XRD) patterns. The results revealed a decrease in the in-plane parameters from 5.036 to 5.026 Å (a contraction of 0.19%) for x = 0–0.5, while the out-of-plane parameters showed a more significant drop from 14.918 to 14.812 Å (a reduction of 0.71%). This indicates that the substitution of Sr^2+^ ions by Ca^2+^ ions has a dominant effect on the out-of-plane direction, while the in-plane parameters remain largely unaffected.

Since the rock-salt layer is the primary contributor to the unit cell and XRD pattern, we consider the average in-plane parameters as the average b-parameter of the rock-salt layer. This leads us to the conclusion that the substitution of Sr^2+^ ions by Ca^2+^ ions does not significantly impact the misfit ratio or the valence of cobalt, as reflected in their transport and thermoelectric properties.

It is also unlikely that the misfit ratio changes due to oxygen vacancies, as all of our samples were sintered under the same conditions and with high oxygen flow.

**Figure 5:**
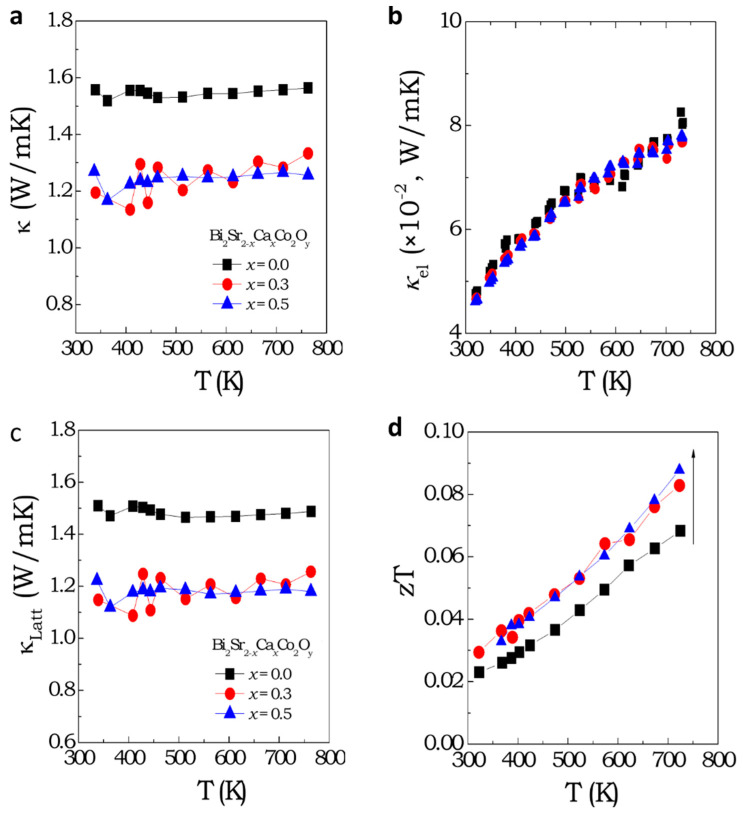
Thermal transport and thermoelectric figure of merit. (**a**) Thermal conductivity as a function of temperature, (**b**) calculated electronic part of the thermal conductivity from the electrical conductivity and Lorentz number by using the Wiedemann-Franz law, (**c**) subtracted lattice part of the thermal conductivity, and (**d**) temperature dependence of the thermoelectric figure of merit of Bi_2_Sr_2-x_Ca_x_Co_2_O_y_ (x = 0, 0.3 and 0.5) samples.

The electrical conductivity of the undoped sample exhibits slight fluctuations around 400 K, as depicted in Figure 4a. These variations could be a result of slight fluctuations in the experimental setup. Nevertheless, similar variations are also observed in the k measurements (Figure 5a), indicating a potential correlation between the two. However, we do not know the exact underlying mechanism behind these fluctuations.

Moreover, the S in the single crystal [38] and polycrystals [30] of BSCO below 300 K shows an interesting behavior. It increases in the temperature range between 3–150 K and then remains constant above 200 K. The temperature-independent S is an indication of the contribution of narrowband (i.e., incoherent charge carriers) to the thermopower [39]. Due to the triangular distortion in the CoO_2_ layer, t_2g_ orbital splits into in-plane Co e^′^_g_ and out-of-plane Co a_1g_ orbitals [23]. The states near E_F_ have a_1g_ symmetry, and electrons undergo hopping transport in the neighbouring Co a_1g_ states [40]. Therefore, for localized interacting charge carriers (at the high-temperature limit, i.e., when bandwidth, W << thermal energy, k_B_T), S is governed by the statistical distribution of charge carriers over available sites, given by Heike’s formula [39] as follows;

(2) $$S=-\frac{k_{B}}{e}\ln\left(\frac{c}{1-c}\right),$$

where, $\;k_{B}$, e, and c are the Boltzmann constant, electron charge, and the ratio of particles (holes in this case) to sites, respectively.

Although the polycrystalline samples in this work did not show any sign of saturation of S above 340 K, a rough estimation of c = 0.22 was obtained from the measured average S = +110 μV/K by using Equation (2). Here, c is directly related to the oxidation state of cobalt. From the framework of Heike’s formula, c can be simplified to the number of charge carriers per unit cell. Therefore, roughly, an oxidation state of +3.22 can be estimated for cobalt, which is slightly less than the reported valence of cobalt in BSCO (of composition Bi_2.1_Sr_2.15_Co_2_O_8+δ_) [41]. Although the possibility of spin and orbital degrees of freedom (β) was proposed to contribute to the S in misfit cobaltates [42,43], recent reports in the thin films of misfit cobaltates showed that the use of β factor overestimates the measured S at 300 K [44]. The temperature-dependence of the PF of BSCO and their alloys are depicted in Figure 4c. The σS^2^ of all the samples are ~1.1 μW/cmK^2^ at room temperature and increased to a maximum value ~1.55 μW/cmK^2^ at 775 K.

Figure 5a depicts κ as a function of the temperature of Bi_2_Sr_2-x_Ca_x_Co_2_O_y_ (x = 0, 0.3 and, 0.5) compounds from 340–750 K. The κ of the parent BSCO polycrystalline pellet is ~ 1.6 W/mK at 340 K, which is lower than the thermal conductivity value reported in a single crystal [21]. Moreover, we observe that k exhibits a very weak temperature-dependence. The low κ of the misfit cobaltates is mainly attributed to the phonon scattering at the CoO_2_ and RS interfaces and the highly ordered RS layer [20]. However, the lower κ for polycrystalline samples reflects the important contribution of phonon scattering at the grain boundaries. In the literature, a large discrepancy in the absolute value of the thermal conductivity of BSCO polycrystals and single crystal is observed, which varies from ~0.8 to ~4 W/mK at 300 K. This variation is attributed to the different methods of sample preparation [19,27], measurement techniques [20,21,45], as well as the sintering process followed before measurements [32]. A comparison of the reported κ at 300 K is presented in Table 1. The temperature-independent nature of κ suggests that the grain boundaries and impurities mainly dominate the phonon scattering in the samples. The measured κ of the parent Bi_2_Sr_2_Co_2_O_y_ at 340 K is comparable with previous reports in polycrystalline samples [29,32] prepared by similar high-temperature solid state reactions and sol-gel methods, respectively.

A drop in thermal conductivity (κ) from 1.6 to 1.2 W/mK was observed at 340 K due to the substitution of Sr atoms with Ca atoms, linked to changes in the crystal structure. Despite typical phonon–phonon scattering in thin film semiconductors at high temperatures [46,47], the polycrystalline nature of the samples limits phonon MFP at grain boundaries, resulting in temperature-independent κ. The addition of dopants with varying ionic radii also reduces thermal conductivity through impurity scattering. It is well known that the measured κ consist of two different parts; one is the electronic part (κ_el_) and the other the lattice part (κ_Latt_). The κ_el_ is directly proportional to the electronic conductivity (σ) according to the Wiedeman-Frentz law. The relation can be mathematically expressed as; κ_el_ = LσT, where L is the Lorentz number. Therefore, κ_Latt_ can be separated from the measured *κ* if L is known. One possibility is to consider the standard value of L, which is ~2.45 × 10^−8^ W/S^−1^K^−2^, and another possibility is to estimate L from measured thermopower data. L is directly related to the scattering factor (ξ) and the reduced Fermi energy (η) by the following mathematical relation [48];

(3) $$L={\left(\frac{k_{B}}{e}\right)}^{2}\left(\frac{{(\xi +7/2)F}_{\xi +\frac{5}{2}}\left(\eta \right)}{{(\xi +3/2)F}_{\xi +\frac{1}{2}}\left(\eta \right)}-{\left(\frac{\left(\xi +\frac{5}{2}\right)F_{\xi +\frac{3}{2}}\left(\eta \right)}{\left(\xi +\frac{3}{2}\right)F_{\xi +\frac{1}{2}}\left(\eta \right)}\right)}^{2}\right),$$

However, η is needed to calculate L from Equation (2). η can be obtained from the measured Seebeck data from Figure 3b by using the single parabolic band model given by;

(4) $$S=\text{±}\frac{k_{B}}{e}\left(\frac{{(\xi +5/2)F}_{\xi +\frac{3}{2}}\left(\eta \right)}{{(\xi +3/2)F}_{\xi +\frac{1}{2}}\left(\eta \right)}-\;\eta \right),\;$$

where,$\;F_{n}\left(\eta \right)$ is nth order Fermi integral: $F_{n}\left(\eta \right)=\int_{0}^{\infty }\frac{\chi^{n}}{{1+e}^{\chi -\eta }}d\chi$ and $\eta =\frac{E_{F}}{k_{B}T}$, where E_F_ is the Fermi energy and k_B_ is the Boltzmann constant. Here, we assume that acoustic phonon is the main scattering mechanism of electrons, i.e.,$\;\xi =-\frac{1}{2}$. Although the Seebeck coefficient was expressed by the single parabolic model (in Equation (4)), the actual electronic band structure of the misfit cobaltates is rather complex (as has been described before). All the above calculations with simple assumptions were carried out only in order to obtain L. The calculated L from the measured *S* is within the range of ~1.61 × 10^−8^ WS^−1^K^−2^. The separated k_el_ is shown in Figure 5b, which is two orders of magnitude lower than the κ_Latt_ part (see Figure 5c). Therefore, the reduced κ at 340 K is mainly due to phonons scattering at the point defects in the RS layer originated from the substitution of Sr ions by Ca. Figure 5d represents the zT value of parent Bi_2_Sr_2_Co_2_O_y_ and Bi_2_Sr_2-x_Ca_x_Co_2_O_y_ (x = 0.3, 0.5) compounds. The highest zT value of 0.09 at 750 K was achieved for the Bi_2_Sr_2-x_Ca_x_Co_2_O_y_ (x = 0.3 and 0.5) samples. A fair comparison of the estimated zT values of our samples with the reported values in the literature is difficult due to that fact that measured thermal conductivity values varies significantly, as described in Table 1.

## 4. Summary

In summary, polycrystalline bulk samples of Bi_2_Sr_2-x_Ca_x_Co_2_O_y_ (x = 0, 0.3 and 0.5) were synthesized by conventional solid-state reactions. The substitution of Sr^2+^ ions by Ca^2+^ ions was confirmed through X-ray diffraction, X-ray photoelectron spectroscopy, and Raman spectroscopy. The isovalent substitutions of Sr^2+^ ions in the BSCO lattice results in a negligible impact on electronic and thermoelectric transport properties, but a significant effect on lattice thermal conductivity due to selective phonon scattering.

This reduction leads to a marked improvement in the overall thermoelectric figure-of-merit. Our findings highlight that selective phonon scattering can play a crucial role in enhancing the thermoelectric performance of misfit layer compounds, such as Bi_2_Sr_2-x_Ca_x_Co_2_O_y_.

## Figures and Tables

**Table 1:** Comparison of the measured thermal conductivity of Bi_2_Sr_2_Co_2_O_y_ polycrystalline samples with the reported values in the literature.

Composition	Crystallinity	Method of Synthesis	κ (W/mK)	T (K)	Sintering Method	Method of Measurement	Reference
Bi_2_Sr_2_Co_2_Oy	Single crystal	Floating zone technique	1.8	300	-	4 probe method in PPMS	Diao, et al. [45]
Bi_2_Sr_2_Co_2_O_y_	Whisker	Sintered precursor (SP) method	2.2 (in-plane)	300	-	Diffusivity	Funahashi, et al. [16]
Bi_2_Sr_2_Co_2_O_y_	Single crystal	traveling-solvent floating-zone method	2.0 (b-axis) 2.4 (a-axis)	300	-	Herman method	Satake, et al. [21]
Bi_2_Sr_2_Co_2_O_y_	Single crystal	Flux method	0.24 (ab-plane)	300	-	Time domain thermoreflectance	Li, et al. [19]
Bi_2-x_Pb_x_Sr_2_Co_2_O_y_	Single crystal	traveling-solvent-floating-zone technique	0.45 (along a-axis) 3.0 (along b & c-axes)	300	-	Steady state method	Terasaki, et al. [20]
Bi_2-x_Pb_x_Sr_2_Co_2_O_y_	Single crystal	optical floating-zone method	4.4 (x = 0) 3.0 (x = 0.35) 2.8 (x = 0.55)	300	-	Closed cycle refrigerator (direct heat pulse)	Hsu, et al. [26]
Bi_2_Sr_2_Co_2_O_y_	Polycrystalline	Solid state heat treatment	0.8–1.4	400	Sintered at 1113 K in air	Laser flash	Funahashi, et al. [29]
Bi_2_Sr_2-x_Ca_x_Co_2_O_y_	Polycrystalline	Sol-gel	3.5 (x = 0) 2.0 (x = 0.3) 2.8 (x = 0.5)	300	Sintered at 1073 K	4 probe method in PPMS	Yin, et al. [30,31]
Bi_2_Sr_2_Co_2_O_y_	Polycrystalline	Sol-gel	1.8 (0 Tesla) 1.6 (4 Tesla) 1.5 (8 Tesla)	300	high magnetic field sintering	4 probe method in PPMS	Huang, et al. [32]
Bi_2_Sr_2-x_Ca_x_Co_2_O_y_	polycrystalline	Solid state reactions at high temperature	1.6 (x = 0) 1.2 (x = 0.3) 1.3 (x = 0.5)	340	Spark plasma sintered	Laser flash	This work

## Data Availability

Raw data can be provided by the corresponding and first author (A.C.) on reasonable request.
